# Identification of azurocidin as a potential periodontitis biomarker by a proteomic analysis of gingival crevicular fluid

**DOI:** 10.1186/1477-5956-9-42

**Published:** 2011-07-28

**Authors:** Young-Jin Choi, Sun-Hee Heo, Jae-Mok Lee, Je-Yoel Cho

**Affiliations:** 1 Department of Biochemistry, School of Dentistry, Kyungpook National University, Daegu, Korea; 2 Department of Periodontology, School of Dentistry, Kyungpook National University, Daegu, Korea

## Abstract

**Background:**

The inflammatory disease periodontitis results in tooth loss and can even lead to diseases of the whole body if not treated. Gingival crevicular fluid (GCF) reflects the condition of the gingiva and contains proteins transuded from serum or cells at inflamed sites. In this study, we aimed to discover potential protein biomarkers for periodontitis in GCF proteome using LC-MS/MS.

**Results:**

We identified 305 proteins from GCF of healthy individuals and periodontitis patients collected using a sterile gel loading tip by ESI-MS/MS coupled to nano-LC. Among these proteins, about 45 proteins were differentially expressed in the GCF proteome of moderate periodontitis patients when compared to the healthy individuals. We first identified azurocidin in the GCF, but not the saliva, as an upregulated protein in the periodontitis patients and verified its increased expression during periodontitis by ELISA using the GCF of the classified periodontitis patients compared to the healthy individuals. In addition, we found that azurocidin inhibited the differentiation of bone marrow-derived macrophages to osteoclasts.

**Conclusions:**

Our results show that GCF collection using a gel loading tip and subsequent LC-MS/MS analysis following 1D-PAGE proteomic separation are effective for the analysis of the GCF proteome. Our current results also suggest that azurocidin could be a potential biomarker candidate for the early detection of inflammatory periodontal destruction by gingivitis and some chronic periodontitis. Our data also suggest that azurocidin may have an inhibitory role in osteoclast differentiation and, thus, a protective role in alveolar bone loss during the early stages of periodontitis.

## Background

Periodontal diseases are a type of inflammatory disease that affect the periodontium surrounding and supporting the teeth and are caused by microorganisms that adhere to and grow on the surfaces of teeth. Most cases of destructive periodontitis are composed of aggressive and chronic periodontitis [1]. Half of the adults in the U.S. suffer from chronic periodontitis, which is the most general form of destructive periodontitis, and about 10% of the population is at a high risk for severe periodontitis [2]. It has been suggested that chronic periodontitis can lead to bone loss and tooth mortality, which might lead to further systemic diseases, e.g., cardiovascular disease and stroke [3,4].

A diagnosis of periodontitis is established by traditionally used indices, e.g., bleeding upon probing and probing depth, which indicate the loss of periodontal tissue attachment to the teeth [5,6]. Additionally, radiography visualizes the loss of periodontal tissue, which supports the diagnosis by determining the amount of bone loss around the teeth [7]. However, these methods are only useful when attachment loss has occurred to some degree. For chair-side and single visits, more reliable biomarkers for periodontitis are needed to provide constant classification and customized treatment and for monitoring periodontal diseases [8,9].

Gingival crevicular fluid (GCF) is bodily fluid that reflects the condition of the periodontium [10]. GCF contains various components that originate by transudation of the serum or inflammatory factors and are derived from the interaction between the bacterial biofilm and the cells of periodontal tissues [11,12]. Additionally, despite a large variation, the amount of GCF tends to increase with the severity of gingival inflammation [13]. In addition to these characterizations, the simple and noninvasive collection of GCF is required for the discovery of periodontitis biomarkers. However, GCF collection using filter paper strips and microcapillary tubes, two generally used methods, have drawbacks such as nonspecific binding of the analyte to paper fibers and the loss of the collected sample, respectively [10].

In previous decades, proteomic analysis has been successfully used for various purposes, such as the identification of binding partner proteins to certain factors and novel proteins derived under specific circumstances. In particular, proteomic technology has become an important tool for the discovery of protein/peptide biomarkers. Proteomic analysis of bodily fluids, e.g., blood, urine, and GCF, is widely used for biomarker discovery because of its easy accessibility for analysis and diagnosis [14].

In this study, we isolated GCF from periodontitis patients and healthy individuals using a gel loading tip. In an effort to discover potential protein biomarkers for periodontitis in GCF, protein samples from the GCF were used for the analysis of the GCF proteome using LC-MS/MS.

## Methods

### Human GCF samples

GCF samples were collected from periodontitis patients and healthy individuals for the identification of periodontitis biomarkers at the Department of Periodontology, School of Dentistry, Kyungpook National University. The standard protocol was approved by the IRB of Kyungpook National University Hospital (IRB NO. 74005 - 830). Informed consent was obtained from all donors.

### Clinical parameters and the establishment of a standard protocol for GCF collection

**Figure 1:**
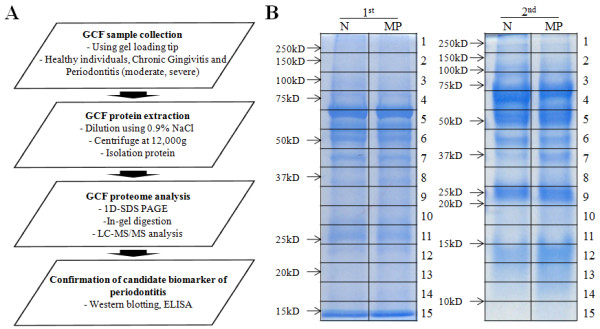
**Schematic diagram of the experimental procedures and 1D SDS gel image after Coomassie staining**. **(A) **GCF samples were collected from healthy individuals and chronic gingivitis, moderate periodontitis and severe periodontitis patients using a gel loading tip. The collected samples were diluted with 0.9% NaCl solution, and the GCF proteins were isolated. Analysis of the GCF proteome was performed using LC-MS/MS. The proteins selected as candidate biomarkers of periodontitis were verified using western blotting and ELISA. **(B) **Pooled samples from 2 sets were separated by 1D SDS-PAGE and stained with Coomassie Brilliant Blue for the visualization of proteins. The stained proteins were excised in 15 pieces for in-gel trypsin digestion. Peptides were isolated from four pooled samples, 2 normal and 2 chronic periodontitis, and analyzed in duplicate by LC-MS/MS (Additional files 1). All subjects were non-smokers and had no other disease indications or other conditions, e.g., diabetes, arteriosclerosis, hepatitis, AIDS, hypertension or pregnancy.

To stratify the chronic gingivitis and chronic periodontitis patients, the clinical periodontal parameters from average 6 sites per individual were assessed at the initial examination for mean probing depth (PD), clinical attachment loss (CAL), bleeding upon probing (BOP), and the gingival index (GI) (Table 1) [15]. According to the criteria and complications, we divided the samples into normal (N), chronic gingivitis (GV), moderate periodontitis (MP), and severe periodontitis (SP) and collected GCF with a sterile gel loading tip (DASLAB, Spain). Each individual sample was collected from the sites of gingival sulcus with same classification of periodontitis according to our criteria. Because of the small volume of collected GCF (about 0.5 to 2 μl per patient), all of the collected samples were directly diluted into 20 μl of 0.9% NaCl solution for the convenience of sample preparation. Diluted samples were centrifuged at 13,000 g (4°C) for 15 min, and the supernatants were transferred to new tubes to remove contaminants such as tissue debris. The transferred samples were used for protein quantification and stored at -70°C until further assay (Figure 1A). Among the 869 GCF samples (161 normal, 229 gingivitis, 296 moderate periodontitis, and 183 severe periodontitis) collected from July 2009 to Aug. 2010, we used GCF samples without complications such as diabetes, arteriosclerosis, hepatitis, AIDS, hypertension, or pregnancy for LC-MS/MS analysis, western blotting, and ELISA.

**Table 1:** Criteria for the classification of collected GCF.

	N	GV	MP	SP
**GI**	**0**	**1**	**2**	**3**
	**Normal gingiva**	**mild inflammation, slight change in color and slight edema, no BOP**	**moderate inflammation, redness, edema, glazing, BOP**	**severe inflammation, marked redness and edema, ulceration, tendency toward spontaneous bleeding**

**PD**	**1 mm**	**≤ 3 mm**	**3 - 7 mm**	**≥ 7 mm**

**CAL**	**0**	**0**	**< 5 mm**	**≥ 5 mm**

**Evidence of bone loss on radiograph**	**-**	**-**	**+**	**+**

### Collection of gingival tissue

Gingival tissue samples were obtained from patients undergoing periodontal surgery at the Department of Periodontology, School of Dentistry, Kyungpook National University. The standard protocol was approved by the IRB of Kyungpook National University Hospital (IRB NO. 74005 - 830). Informed consent was obtained from all donors. Gingival tissues were classified as normal (N), gingivitis (GV), moderate periodontitis (MP), or severe periodontitis (SP) according to criteria such as the gingival index considered immune reaction, probing depth, clinical attached loss, and bone loss on radiograph (Table 1). For western blotting, gingival tissues were isolated from 2 healthy individuals (N), 3 with chronic gingivitis (GV), 3 with moderate periodontitis (MP), and 3 with severe periodontitis (SP).

### LC-ESI-MS/MS analysis following in-gel digestion

We selected GCF samples collected from 11 normal (N) and 12 moderate periodontitis (MP) patients without systemic complications (Additional files 1) for analysis of the GCF proteomes using LC-MS/MS. Twenty micrograms of pooled samples was separated by 1D gel electrophoresis. Gels were stained with Bio-Safe Coomassie Stain solution (Bio-Rad, Hercules, CA) for 1 h and destained by incubation in ddH_2_O. In-gel tryptic digestion was performed as previously described [16]. Briefly, protein bands were excised from Coomassie-stained gels and destained. Disulfide bonds were reduced by treatment with 5 mM Dithiothreitol (DTT)/25 mM ammonium bicarbonate (Sigma-Aldrich, St. Louis, MO) at 60°C for 30 min, followed by alkylation with 55 mM iodoacetamide (Sigma-Aldrich, St. Louis, MO) at RT for 30 min. The gel pieces were dehydrated in 100% acetonitrile (ACN) (Sigma-Aldrich, St. Louis, MO) and dried. They were then subsequently rehydrated in 10 μl of 25 mM ammonium bicarbonate buffer containing 20 μg/ml modified sequencing grade trypsin (Roche, Mammheim, Germany) and incubated overnight at 37°C. The tryptic peptide mixture was eluted from the gel with 0.1% formic acid (Fluka, Switzerland).

LC-MS/MS analysis was performed using a Thermo Finnigan ProteomeX workstation LTQ linear ion trap MS/MS (Thermo Electron, San Jose, CA, USA) equipped with NSI sources (San Jose, CA) as previously reported [17,18]. Briefly, 12 μl of peptide sample from the in-gel digestion was injected and loaded onto a peptide trap cartridge (Agilent, Palo Alto, CA). Trapped peptides were eluted onto a 10-cm reversed-phase PicoFrit column packed in-house with 5 μm, 300 Å pore size C18 and then separated by gradient elution. The mobile phases consisted of H_2_O (A) and ACN (B), and both phases contained 0.1% (v/v) formic acid. The gradient started at 2% B, reached 60% B in 50 min, 80% B in the next 5 min, and 100% A in the final 15 min. Data-dependent acquisition (m/z 400-1800) was enabled. Each survey MS scan was followed by five MS/MS scans within 30 sec with the dynamic exclusion option on. The dynamic exclusion option is a tool for the exclusion of basal peaks to increase proteome coverage by removing background noise in MS/MS peaks.

### Data analysis

Tandem mass spectra were extracted and charge state deconvoluted and deisotoped using Sorcerer 3.4 beta2 (Sorcerer software 3.10.4, Sorcerer Web interface 2.2.0 r334 and Trans-Proteomic Pipeline 2.9.5), as previously reported [18]. All MS/MS samples were analyzed using SEQUEST (Thermo Finnigan, San Jose, CA; version v.27, rev. 11). SEQUEST was set to search the IPI Human 3.49 database (IPI ver. 3.49, 74,017 entries) assuming semiTrypsin as the digestion enzyme. SEQUEST search parameters were set to a fragment ion mass tolerance of 1.0 Da and a parent ion tolerance of 1.5 Da. Oxidation of methionine and iodoacetamide modification of cysteine were specified as fixed modifications. Scaffold (version Scaffold-02_04_00, Proteome Software Inc., Portland, OR) calculates the quantitative value number by normalizing the spectral counts across our Scaffold experiment. For improved false-positive statistics, the decoy option was selected during the data search process in the Sorcerer program, which improves the quality of the results by reducing the effects of noise. Comparative data analysis using Scaffold was subsequently used to validate MS/MS-based peptide and protein identifications. Protein identifications were accepted if they could be established at a confidence level greater than 95.0% probability, as specified by the Peptide Prophet algorithm [19]. Protein probabilities were assigned by the Protein Prophet algorithm [20]. Proteins that contained similar peptides and that could not be differentiated based on the MS/MS analysis alone were grouped to satisfy the principles of parsimony. The peptide false positive (FPR) rate was calculated using the Scaffold software. For each charge state, incorrect assignments were tabulated to calculate the FPRi = [(#Assigned Incorrect at 95% probability)/(Total# Incorrect Assigned)]*100, with *i *being the charge state. The assignment is considered correct if it is associated with a protein that has a 95% probability, according to the Protein Prophet algorithm, and if a minimum of 2 peptides are matched with the protein sequence, each with a 95% probability, based on the Peptide Prophet algorithm. After identifying the proteins, each dataset was used for a subtractive analysis by quantitative value, which normalizes the spectral counts in the Scaffold program.

### Western Blot

Western blots were performed as previously reported [21]. Briefly, 5 μg of gingival tissue was separated by 15% SDS-PAGE (Elpis-Biotech, Korea) for azurocidin. After electrophoresis, the proteins were transferred to PVDF membrane (Roche, USA), which was incubated with anti-human azurocidin antibody (Santa Cruz, CA) (1:1000) and β-actin antibody (Abcam, Cambridge, MA) (1:2000), followed by an anti-rabbit IgG antibody (1:2000) for azurocidin and an anti-mouse IgG antibody (1:2000) for β-actin.

### ELISA

For the verification of azurocidin expression in GCF, samples were selected from 31 N, 36 gingivitis (GV), 59 MP, and 30 severe periodontitis (SP) patients (Additional files 2). Selected samples were collected from non-smokers and subjects having no complications (Additional files 2). The ELISA (Cusabio, China) for azurocidin was performed following the manufacturer's instructions. Briefly, samples were added in a well and incubated for 2 h (37°C). The liquid from each well was removed, and 100 μl of biotin-antibody working solution was then added to each well for 1 h (37°C). The mixture was removed and washed with the provided buffer (350 μl) using a multi-channel pipette. After washing, HRP-avidin working solution was added to each well and incubated for 1 h (37°C). After washing, TMB substrate was added and incubated for 30 min (37°C), and the reaction was stopped by adding 50 μl of Stop Solution to each well. The optical density of each well was determined using a microplate reader set to 450 nm.

### Osteoclast differentiation and TRAP staining

Bone marrow cells were isolated from the tibiae and femora of 5-week-old BALB/c mice and cultured with α-MEM containing 10% FBS in a humidified incubator (5% CO_2_) at 37°C. After 24 h, non-adherent cells were centrifuged to obtain bone marrow-derived macrophage (BMM) cells and incubated for use as adherent BMM for 3 days in the presence of 20 ng/ml macrophage colony stimulating factor (M-CSF) (Peprotech, USA). For the osteoclastogenesis experiments, BMM cells were cultured in the presence of 20 ng/ml RANKL (R&D, USA) and 20 ng/ml M-CSF with azurocidin (R&D, USA) at the indicated concentration. TRAP staining was performed according to the manufacturer's instructions (Sigma, USA) [22].

### Statistical analysis

The data are presented as the means ± SE. The statistical significance of the results was assessed using one-way ANOVA, followed by Student's t-test for unpaired samples. All p-values were derived from two-tailed statistical tests, and a p-value < 0.05 was regarded as statistically significant.

## Results

### General strategy and GCF protein visulalization by Coomassie staining

To analyze the GCF proteomes by LC-MS/MS, we selected 11 normal (N) and 12 moderate periodontitis (MP) patients without systemic complications (Additional files 1). For a representative group of periodontitis, MP samples were selected for LC-MS/MS analysis. Proteins in the pooled samples were separated by SDS-PAGE. The patterns of SDS-PAGE-separated proteins were visualized by Coomassie staining (Figure 1B). In the region near 37 kDa (8^th ^band of the first pool and 7^th ^band of the second pool), a protein band was stained more intensely in MP compared to N.

### Analysis of the GCF proteome by LC-MS/MS

Proteins from 4 different pooled GCF samples were identified by the duplicate LC-MS/MS analysis of each (total of 8 analyses). By searching the human IPI databases with a protein identification criteria of at least 2 peptides, a total of 305 proteins were identified from the combined analysis of both the N and MP samples (Additional files 3). These data revealed that the reverse peptide hit rate was 0.94 ± 0.1% of total peptide hits using the decoy protein database. After normalization of the spectral counts by total ion currents in the Scaffold program, we calculated the average spectral counts of 4 analyses (duplicate analysis of two sets of experiments) in each N and MP sample and then calculated the fold ratio of MP vs. N (MP/N). The results revealed that 25 proteins demonstrated a 2-fold or higher ratio of average spectral counts in the MP samples (Table 2), and 20 proteins demonstrated a 2-fold or higher ratio of average spectral counts in the N samples (Table 3). Additionally, 45 proteins were classified based on biological process, cellular component, and molecular function using the UniProt database (Tables 2 and 3). By searching the NCBI bacterial database, proteins from bacteria known to cause periodontitis were also identified (Additional files 4).

**Table 2:** List of upregulated proteins in periodontitis GCF.

#	Identified Proteins	Accession Number	Biological process	Cellular component	Molecular function	Molecular Weight	N	MP	MP/N fold	N/MP fold
1	Catalase	IPI00465436	hydrogen peroxide removal	peroxisome	mitogen oxidoreductase peroxidase	60 kDa	0.3 ± 0.4	3.0 ± 1.4	12.0	0.1
2	Adenylyl cyclase-associated protein 1	IPI00008274	signal transduction	cell membrane	actin binding	52 kDa	0.5 ± 0.7	5.8 ± 6.0	11.5	0.1
3	Plastin-2	IPI00010471	T cell activation	cell junction	calcium ion binding	70 kDa	2.0 ± 0.7	15.0 ± 12.7	7.5	0.1
4	Alpha-actinin-1	IPI00013508	negative regulation of cellular component	cytosol	actin binding	103 kDa	1.5 ± 0.7	10.3 ± 13.1	6.8	0.1
5	Fibrinogen beta chain	IPI00298497	platelet activation	external side of plasma membrane	cell surface binding	56 kDa	0.8 ± 0.4	4.8 ± 1.8	6.3	0.2
6	Hornerin	IPI00398625	keratinization		developmental protein	282 kDa	1.0 ± 1.4	5.5 ± 4.2	5.5	0.2
7	Isoform 1 of Myosin-9	IPI00019502	cell shape	cytosol	motor protein	227 kDa	4.8 ± 3.9	23.5 ± 26.2	4.9	0.2
8	Myeloblastin	IPI00027409	collagen degradation		serine protease	28 kDa	2.3 ± 0.4	11.0 ± 2.8	4.9	0.2
9	Azurocidin	IPI00022246	chemotaxis	azurophil granule	antimicrobial	27 kDa	2.0 ± 0.7	9.3 ± 1.8	4.6	0.2
10	Isoform 1 of Fibrinogen alpha chain	IPI00021885	blood coagulation	Secreted	cell surface binding	95 kDa	0.8 ± 1.1	3.0 ± 2.1	4.0	0.3
11	Leukocyte elastase	IPI00027769	inflammatory response	secretory granule	Serine protease	29 kDa	5.0 ± 4.2	16.0 ± 3.5	3.2	0.3
12	Matrix metalloproteinase-9	IPI00027509	collagen degradation	extracellular matrix	metalloprotease	78 kDa	3.8 ± 3.9	10.5 ± 12.7	2.8	0.4
13	HBD Hemoglobin subunit delta	IPI00473011	oxygen transport	hemoglobin complex	oxygen binding	16 kDa	34.8 ± 7.4	94.8 ± 63.3	2.7	0.4
14	Coronin-1A	IPI00010133	innate immune response	cytoplasm	actin filament binding	51 kDa	5.0 ± 2.8	13.0 ± 13.4	2.6	0.4
15	Rho GDP-dissociation inhibitor 2	IPI00003817	immune response	cytoplasm	GTPase activator	23 kDa	5.0 ± 3.5	12.0 ± 4.9	2.4	0.4
16	Keratin, type II cytoskeletal 5	IPI00009867	assembly	intermediate filament	structural constituent	62 kDa	9.8 ± 11.0	21.8 ± 13.1	2.2	0.4
17	Macrophage-capping protein	IPI00027341	assembly	cytoplasm nucleus	actin binding	39 kDa	1.8 ± 2.5	3.8 ± 1.8	2.1	0.5
18	HBA2 Hemoglobin subunit alpha	IPI00410714	oxygen transport	hemoglobin complex	oxygen binding	15 kDa	49.8 ± 20.9	105.5 ± 66.5	2.1	0.5
19	Keratin, type II cytoskeletal 1	IPI00220327	fibrinolysis	intermediate filament	structural constituent	66 kDa	93.0 ± 79.2	196.5 ± 193.0	2.1	0.5
20	Annexin A3	IPI00024095	defense response to bacterium	phagocytic vesicle	calcium-dependent phospholipid binding	36 kDa	4.5 ± 4.2	9.5 ± 0.7	2.1	0.5
21	Hemoglobin subunit beta	IPI00654755	oxygen transport	hemoglobin complex	hypotensive agent	16 kDa	74.5 ± 7.1	155.8 ± 83.8	2.1	0.5
22	Keratin, type I cytoskeletal 9	IPI00019359	organization	intermediate filament	structural constituent	62 kDa	31.0 ± 22.6	64.8 ± 63.3	2.1	0.5
23	Keratin, type II cytoskeletal 2 epidermal	IPI00021304	keratinization	intermediate filament	structural constituent	66 kDa	32.3 ± 39.2	65.0 ± 72.1	2.0	0.5
24	Actin-related protein 2	IPI00005159	cellular component	cytoplasm	actin binding	45 kDa	0.8 ± 1.1	1.5 ± 0.7	2.0	0.5
25	S100-A9	IPI00027462	chemotaxis	extracellular region	calcium ion-binding protein	13 kDa	44.8 ± 1.1	88.5 ± 1.4	2.0	0.5

**Table 3:** List of downregulated proteins in periodontitis GCF.

#	Identified Proteins	Accession Number	Biological process	Cellular component	Molecular function	Molecular Weight	N	MP	MP/N fold	N/MP fold
1	Cystatin-A	IPI00032325	keratinocyte	cytoplasm	cysteine-type endopeptidase inhibitor activity	11 kDa	4.5 ± 3.5	2.3 ± 1.1	0.5	2.0
2	Thymidine phosphorylase	IPI00292858	chemotaxis		glycosyltransferase	50 kDa	7.5 ± 2.8	3.8 ± 0.4	0.5	2.0
3	Cystatin-B	IPI00021828		cytoplasm nucleus	protease inhibitor	11 kDa	11.0 ± 5.7	5.3 ± 1.1	0.5	2.1
4	Cystatin-SN	IPI00305477		secreted	cysteine-type endopeptidase inhibitor activity	16 kDa	13.5 ± 0.7	6.3 ± 2.5	0.5	2.2
5	IGKV3D-15 Myosin-reactive immunoglobulin light chain variable region	IPI00549330				13 kDa	6.3 ± 0.4	2.8 ± 0.4	0.4	2.3
6	Nucleoside diphosphate kinase B	IPI00026260	transcription regulation	cytoplasm nucleus	kinase	17 kDa	6.0 ± 3.5	2.5 ± 1.4	0.4	2.4
7	14-3-3 protein epsilon	IPI00000816	host-virus interaction	cytoplasm	histone deacetylase binding	29 kDa	9.0 ± 1.4	3.5 ± 3.5	0.4	2.6
8	Prolactin-inducible protein	IPI00022974		secreted	actin binding	17 kDa	15.0 ± 12.0	5.3 ± 6.0	0.4	2.9
9	alpha-2-glycoprotein 1, zinc	IPI00166729	immune response	secreted	transmembrane transporter activity	34 kDa	13.0 ± 2.8	4.5 ± 2.8	0.3	2.9
10	Polymeric immunoglobulin receptor	IPI00004573		membrane secreted	protein binding	83 kDa	76.0 ± 34.6	25.3 ± 31.5	0.3	3.0
11	Lipopolysaccharide-responsive and beige-like anchor protein	IPI00002255		cell membrane	protein binding	319 kDa	4.8 ± 4.6	1.5 ± 0.7	0.3	3.2
12	IGL@ protein	IPI00555945				25 kDa	69.0 ± 27.6	21.5 ± 13.4	0.3	3.2
13	Heat shock protein beta-1	IPI00025512	stress response	cytoplasm cytoskeleton nucleus	identical protein binding	23 kDa	7.8 ± 1.1	2.3 ± 1.8	0.3	3.4
14	Glutathione S-transferase P	IPI00219757	anti-apoptosis	cytoplasm	glutathione transferase activity	23 kDa	34.5 ± 30.4	10.0 ± 9.2	0.3	3.5
15	S100-A2	IPI00019869	endothelial cell migration		calcium ion binding	11 kDa	4.5 ± 0.7	1.3 ± 0.4	0.3	3.6
16	Glutathione transferase omega-1	IPI00019755		cytoplasm	glutathione transferase activity	28 kDa	4.0 ± 1.4	1.0 ± 0.7	0.3	4.0
17	Carbonyl reductase [NADPH] 1	IPI00295386	oxidation reduction	cytoplasm	oxidoreductase	30 kDa	7.8 ± 3.2	1.3 ± 1.1	0.2	6.2
18	CRISP3 cDNA FLJ75207	IPI00004798		extracellular region		29 kDa	7.0 ± 7.8	1.0 ± 1.4	0.1	7.0
19	Isoform 1 of Serpin B3	IPI00022204		cytoplasm	serine protease inhibitor	45 kDa	4.5 ± 2.1	0.5 ± 0.0	0.1	9.0
20	Aldo-keto reductase family 1 member B10	IPI00105407	oxidation reduction	cytoplasm	oxidoreductase	36 kDa	4.8 ± 3.2	0.3 ± 0.4	0.1	19.0

### Verification of azurocidin expression in GCF using ELISA

**Figure 2:**
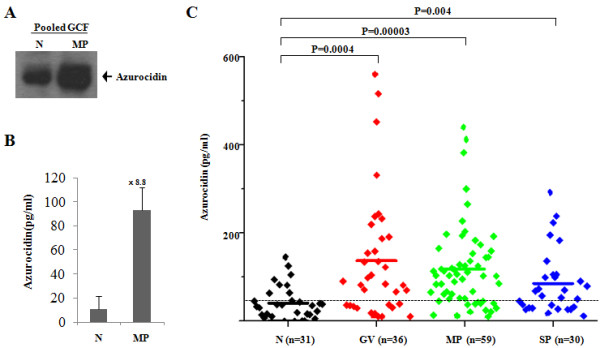
**Verification of azurocidin expression in the GCF samples**. Azurocidin expression was confirmed in the GCF samples used for proteomic analysis by western blot **(A) **and the ELISA assay **(B)**. **(C) **Azurocidin expression was further confirmed by ELISA in 156 individual GCF samples: 31 healthy individuals (N), 36 chronic gingivitis (GV), 59 moderate periodontitis (MP), and 30 severe chronic periodontitis (SP) patients (Additional files 2). All subjects were non-smokers and had no other disease indications or other conditions, e.g., diabetes, arteriosclerosis, hepatitis, AIDS, hypertension or pregnancy. Black diamond, Individuals; **-**, Average; ---, Cutoff. After the cutoff was set at 50 pg/ml, the sensitivity and specificity of azurocidin for the periodontitis patients were calculated (Table 4).

**Figure 3:**
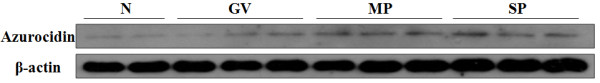
**Azurocidin expression in gingival tissues by western blot**. Protein expression levels of azurocidin were confirmed in gingival tissue using western blotting. Gingival tissues were sorted by the stage of chronic gingivitis and periodontitis. The gingival tissues used for the confirmation of azurocidin expression were from 2 healthy individuals (N) and 3 chronic gingivitis (GV), 3 moderate periodontitis (MP), and 3 severe chronic periodontitis (SP) patients. The detected sizes of azurocidin were approximately 27 kDa. After loading 5 μg of protein, anti-human azurocidin and anti-β-actin antibodies were used for detection. Overall, azurocidin expression was higher in periodontitis patients as compared to healthy individuals.

Among the proteins upregulated in periodontitis, azurocidin was first selected to validate on a larger scale because it is known to be secreted by neutrophils upon inflammatory challenges and because it is an attractant for monocyte infiltration into inflamed tissues [23,24]. Thus, azurocidin levels were measured by western blotting and ELISA in the same samples used for LC-MS/MS analysis (Figures 2A and 2B). Using an ELISA, azurocidin was detected at an 8.8-fold higher level (10.6 pg/ml in N and 92.5 pg/ml in MP) in the GCF of MP compared to N. This dramatic increase may be due in part to the higher sensitivity of ELISA. To avoid prejudice against a small sample size, we performed an ELISA assay of azurocidin with 31 N, 36 gingivitis (GV), 59 MP, and 30 severe periodontitis (SP) patients (Additional files 2), and the average levels of azurocidin in each sample were 40.8 pg/ml, 136.6 pg/ml, 118.3 pg/ml, and 85.3 pg/ml, respectively. With a 50 pg/ml cutoff, the specificity is 74.2%, and the sensitivities are 66.7% in GV, 76.3% in MP, and 60.0% in SP patients (Figure 2C, Table 4). Additionally, azurocidin was also expressed at a higher level in the gingival tissue of periodontitis patients compared to healthy controls in the western blotting assay (Figure 3). The detected sizes of azurocidin were approximately 27 kDa.

**Table 4:** Comparison of studies that have reported on the identification of the GCF proteome.

	N (n = 31)	GV (n = 36)	MP (n = 59)	SP (n = 30)
**Average (pg/ml)**	40.8	136.6	118.3	85.3

**Specificity (%)**	74.2	-	-	-

**Sensitivity (%)**	-	66.7	76.3	60.0

### Effect of azurocidin on osteoclastogenesis

**Figure 4:**
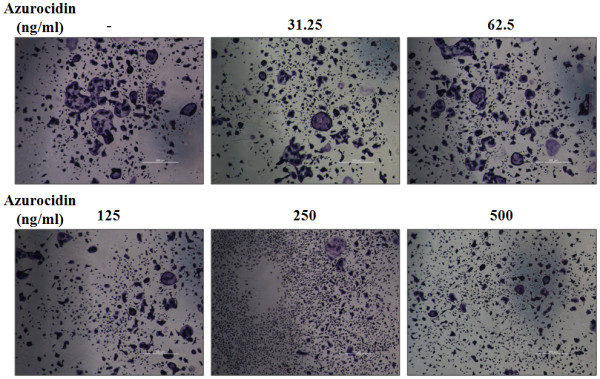
**Inhibition of osteoclast differentiation by azurocidin**. Mouse bone marrow monocytes (BMM) isolated from BALB/c mice were incubated in media containing RANKL (20 ng/ml) plus M-CSF (20 ng/ml) with the given concentration of azurocidin for 3 days. Osteoclast differentiation was evaluated by TRAP staining. The fused osteoclast cells revealed by TRAP staining were decreased with increasing dosages of azurocidin.

Severe periodontitis (SP) results in alveolar bone loss, but not during the early stages of gingivitis. It has been suggested that the regulatory factors of the immune response can affect bone metabolism. Thus, we tested if azurocidin could affect osteoclast differentiation. On day 3, TRAP staining was performed on azurocidin-treated cultures. Osteoclast differentiation of BMM was decreased by the azurocidin treatment (Figure 4), which suggests that high levels of azurocidin at an early stage may have a protective role in osteoclast-activated alveolar bone loss.

## Discussion

In this study, using biologically-independent duplicate experiments of an analytically duplicate LC-MS/MS analysis of the GCF from healthy individuals (N) and moderate periodontitis (MP) patients, 305 proteins were identified. Of these proteins, 45 were significantly up- or downregulated in the GCF of MP patients. We collected 869 GCF samples from patients using a sterile gel loading tip. Among these, we used GCF samples from patients without complications such as diabetes, arteriosclerosis, hepatitis, AIDS, hypertension, or pregnancy for LC-MS/MS analysis, western blotting, and ELISA. We also validated that the azurocidin levels were elevated in periodontitis patients by ELISA in about 150 samples from stratified periodontitis samples and healthy controls.

In our study, for GCF collection, we tried several methods including paper and 26 gauge needles; however, we found that fine gel loading tips are a useful and convenient method for the collection of as much GCF as possible, which resulted in a shortened collection time, a decreased loss of isolated volume and a reduction in the inconvenience/hostility of patients during collection. We identified 305 proteins from the analysis of the GCF proteome by GeLC-MS/MS and also first detected azurocidin as a component of GCF (Additional files 3). In addition, we showed that azurocidin has an effect on the osteoclastogenesis of BMM. Proteins from various bacteria were also identified by searching the LC-MS/MS data against bacterial databases (Additional files 4). However, we could not find significant differences in the bacterial proteomes of the GCF of N and MP patients. For the further analysis of bacterial proteins, more classified samples and an efficient isolation method for bacterial proteins are needed.

Genomic and proteomic studies were performed to discover biologically-regulated signatures in periodontitis patients. Recently, the profiling of periodontitis-related molecules was reported. In 2008, a transcriptome analysis of gingival tissue was performed using 54,675 probe sets in 64 healthy sites and 183 inflamed sites [5]. The study reported 12,744 differentially expressed probe sets, which classified 16 groups related to functions such as apoptosis, the antimicrobial humoral response, and antigen presentation, as determined by gene ontology analysis. There were also proteomic studies on GCF itself. The GCF of a healthy donor was collected using sterile blotting paper cones, and serum albumin, α-defensins 1-4, cystatin A, statherin, and basic PB salivary peptide were detected in the acidic extracts of GCF using HPLC-ESI-MS [25]. In another study, GCF samples collected from inflamed periodontal sites using sterile glass microcapillary tubes were analyzed using MALDI-TOF/TOF MS and LC-ESI-MS/MS. Among the 66 proteins identified in human GCF, 43 proteins, which included actin, profilin, cofilin, and gelsolin, were first reported in GCF [26]. In a recent study, 40 GCF samples were collected from 5 healthy individuals and 5 aggressive periodontitis patients using sterile periopaper strips and analyzed by LC-MS, a label-free mass spectrometry. Among the 154 identified proteins, annexin-1 was expressed at a 5-fold higher level in healthy individuals, and L-plastin (plastin-2/LCP1) was detected only in periodontitis patients [27].

In accordance with a previous report, myeloperoxidase, myosin 9, annexin A3, profilin-1, L-plastin (plastin-2/LCP1), S100A8, and S100A9 were detected at higher levels in chronic periodontitis patients compared to healthy individuals. The expression of cystatin-B was decreased in periodontitis patients compared to healthy controls [27]. Although cornifin-A and S100-P were identified by small peptide hits, S100-P was upregulated and cornifin-A was downregulated in the GCF of periodontitis patients compared to healthy individuals, in both our study and the previous study [27]. Additionally, we identified three proteins, azurocidin, neutrophil elastase 2 (ELA2), and myeloperoxidase (proteinase 3), that are known to be found in azurophil granules, the specialized lysosomes of neutrophils. These three proteins were consistently detected at higher levels in the GCF of chronic periodontitis patients (Table 2). Azurocidin, ELA2, and myeloperoxidase are located in a cluster on chromosome 19 and are constituents of azurophil granules during neutrophil differentiation [28].

Azurocidin is also known as heparin-binding protein (HBP) or cationic antimicrobial protein of 37 kDa (CAP37) [29]. Azurocidin, a member of the serine proteases, is homologous to cathepsin G, elastase, and proteinase 3 (PR-3; myeloblastin) but is known to lack proteolytic activity [30,31]. Azurocidin was first identified as a cationic antimicrobial protein (CAP) in human polymorphonuclear granulocyte (PMN) granules and exhibits an antimicrobial activity against Gram-negative bacteria (*Salmonella typhimurium*), Gram-positive bacteria (*Staphylococcus aureus*) and fungi (*Candida albicans*) [29,32,33]. Recombinant azurocidin induces the release of interleukin-6 and tumor necrosis factor-alpha (TNF-α) from human monocytes upon lipopolysaccharide (LPS) stimulation [34]. During the extravasation cascade of PMN, azurocidin serves as an alarmin and is released from infection sites to activate inflammatory cells [23].

In this study and for the first time, we identified azurocidin in GCF as a periodontitis biomarker. Through mass spectrometry, we determined that the azurocidin level is highly elevated in the GCF of chronic periodontitis patients (Table 2), and we also confirmed these results in different stages of periodontitis using an ELISA analysis (Figure 2C). Azurocidin levels are higher in GV and MP, compared to SP, and might serve as a promising biomarker for the development of the early diagnosis of periodontitis.

Periodontitis is a disease that affects the immune system and results in alveolar bone loss at its severe stages. Evidence has accumulated on the crosstalk between the immune and bone systems [35-38]. Immune response cytokines, such as TNF-α, interleukin, and interferon, affect the differentiation and activity of osteoclasts and bone resorption. In addition, immune cells produce and release key factors (RANKL and M-CSF) for osteoclastogenesis. In regards to this aspect, we have shown the inhibitory effect of azurocidin on osteoclastogenesis from BMM (Figure 4). Thus, it is suggested that azurocidin elevation during gingivitis may have a protective effect on alveolar bone during the early stages of periodontitis. In SP patients, however, the reduced level of azurocidin may lose its protective effect, which may lead to increased alveolar bone loss during later stages of periodontitis.

## Conclusions

Our GCF proteomic study suggests that GCF collection using a gel loading tip followed by LC-MS/MS analysis is a useful method for analyses of periodontitis GCF biomarkers. Our data have revealed that azurocidin may be a potential biomarker candidate for the early detection of periodontal destruction by the inflammation in gingivitis and some chronic periodontitis based on the mass spectrometry, western blot and ELISA data using GCF samples. In addition, we have shown that azurocidin may have an inhibitory role on osteoclast differentiation, which suggests that azurocidin may decrease alveolar bone loss during periodontitis.

## Abbreviations

CAP37: cationic antimicrobial protein of 37 kDa; CAPs: cationic antimicrobial proteins; HBP: heparin-binding protein; GCF: gingival crevicular fluid; LPS: lipopolysaccharide; PMN: polymorphonuclear granulocyte; PPD: probing pocket depth; BOP: bleeding on probing; GI: gingival index; GV: gingivitis; MP: moderate periodontitis; SP: severe periodontitis.

## Competing interests

The authors declare that they have no competing interests.

## Authors' contributions

JC was responsible for planning and designing the study, data interpretation and revising the manuscript. YC performed the experiments and the data analysis and wrote the manuscript. SH participated in designing the experiments and writing the manuscript. JL was responsible for the collection of the GCF and gingival tissues. All authors read and approved the final manuscript.

## Supplementary Material

Additional file 1 **Information for samples used for the identification of the GCF proteome using LC-MS/MS**. Click here for file

Additional file 2 **Information for samples used for the ELISA assay of azurocidin**. Click here for file

Additional file 3 **List of 305 proteins identified from the combined analysis of both N and MP samples**. Click here for file

Additional file 4 **Information about the identified bacterial protein in GCF that was obtained by searching the NCBI database for bacteria known to cause periodontitis**. Click here for file
